# Editorial: Pharmacological and behavioral effects of added flavorants on tobacco addiction

**DOI:** 10.3389/fnins.2022.1100476

**Published:** 2022-12-05

**Authors:** Robert J. Wickham, Brandon J. Henderson, Asti B. Jackson, Nadine Kabbani

**Affiliations:** ^1^Department of Psychology, Lafayette College, Easton, PA, United States; ^2^Department of Biomedical Sciences, Joan C. Edwards School of Medicine, Marshall University, Huntington, WV, United States; ^3^Department of Psychiatry, Connecticut Mental Health Center, Yale University, New Haven, CT, United States; ^4^School of Systems Biology and Interdisciplinary Neuroscience Program, George Mason University, Fairfax, VA, United States; ^5^Center for Biomedical Science Policy, George Mason University, Fairfax, VA, United States

**Keywords:** menthol, ENDS, tobacco, nicotine, addiction, flavor

## Introduction

The chronic use of tobacco products, especially combustible cigarettes, is a leading cause in preventable deaths worldwide. The rising trend in the use of electronic nicotine delivery systems (ENDS), is now a growing public health problem in the United States and elsewhere. Thus, while it is established that nicotine is the primary psychoactive component in tobacco products, additives such as flavors or flavor solvents appear to have their own impacts on tobacco product use and the health of people who use tobacco products. Menthol for example, is the most widely studied flavorant, and published works show that changes to nicotine's pharmacokinetic and pharmacodynamic effects attributable to menthol may explain poorer cessation outcomes in individuals who use mentholated products (reviewed elsewhere in Wickham, 2020). One (of several) of the ways in which menthol may influence tobacco smoking is by masking some of the aversive chemosensory effects of smoking including the bitter taste, irritation, pain, and coughing associated with tobacco product use. In this regard, the addition of menthol is found to improve the overall experience of people who use tobacco products.

Based on this knowledge, it has been suggested that tobacco companies have optimized the level of menthol in mentholated tobacco products to increase tobacco use. Indeed, individuals who start smoking through the use of mentholated cigarettes are found to develop a strong, life-time preference for smoking mentholated cigarettes. Less is known, however, about the impact of other flavorants or additives on smoking behavior and on the use of non-combustible tobacco products such as ENDS. Research suggests that the use of flavored ENDS promotes combustible tobacco product use in young adults (reviewed elsewhere in Zhang et al., 2021). To further explore the impact of added flavors on tobacco product use, we organized this Special Issue of *Frontiers in Neuroscience/Neuropharmacology* consisting of two themes.

## Theme 1: Individual differences in menthol and nicotine preference

Individual preference for menthol and nicotine can vary from person to person and in the context of preclinical studies, strain to strain. The most commonly used mouse strain for animal behavioral studies is the C57BL/6J strain, and this strain shows some levels of dose-dependent oral nicotine preference relative to water. The C57BL/6J strain has also been used to demonstrate menthol's ability to elevate oral nicotine consumption. However, Akinola et al., addressed whether menthol's masking effects and subsequent increase in oral nicotine consumption might still also impact non-nicotine preferring mice, such as the DBA/2J strain. Unlike C57BL/6J, the authors found that menthol did not increase the oral consumption of nicotine within DBA/2J, suggesting the potential for strain-specific genetic differences in menthol-related nicotine effects and elevated consumption. While not yet clear, it is possible that menthol did not mask some of the aversive chemosensory effects of nicotine consumption within the DBA/2J strain.

Humans that smoke cigarettes display distinct preferences between menthol and non-menthol cigarettes, largely based on their cigarette smoking history. In a study by Gueorguieva et al., plasma menthol-glucuronide (MG, a metabolite of menthol) detection was used to predict the physiological and subjective effects of cigarette smoking through a comparison of individuals who smoke menthol and those that smoke non-menthol tobacco products. In this study, subjects smoked a 0, 0.5%, or a 3.2% non-nicotine e-cigarette, while intravenous nicotine was administered simultaneously. During this period, plasma MG levels and subjective ratings of e-cigarette smoking and physiological responses were measured. The authors found that plasma MG levels were not predictive of the subjective effects of intravenous nicotine, the urge to smoke cigarettes, or heart rate changes. Plasma MG levels however did predict the subjective experience of “cooling”—but only in those who smoke menthol cigarettes. Taken together studies by Akinola et al. and Gueorguieva et al. indicate that individual differences, genetic and/or experience-based, impact the role of menthol in nicotine intake or tobacco cigarette smoking experience.

## Theme 2: Chemosensory effects of non-menthol flavors

While menthol is one of the most popular flavors added to tobacco products, including ENDS, there are nearly limitless permutations of flavors and flavor combinations available. In a review by Johnson et al., the authors examine how solvents used to dissolve flavors alone can influence the chemosensory experience of smoking. For example, they report that many e-cigarette blogs and websites discuss varying the ratio of propylene glycol (PG) and vegetable glycerin (VG), two common solvents for flavor additives in ENDS, to influence different chemosensory effects of smoking (greater throat hit, more cloud production, etc.). Additionally, they propose that unlike menthol, various appetitive flavors, like green apple or cherry, can improve the overall experience of e-cigarettes even without the masking of the aversive properties of nicotine (see Jackson et al., 2021 for example). A mini-review by Hayes and Baker proposes that flavors can interact with one another in ways that may participate in tobacco product use. Here, concepts such as mixture suppression, where flavor perception of two mixtures is blunted relative to in isolation, and cross-modal modulation, where one sensory modality can alter the perception of another, may contribute to changes in e-cigarette use in ways not yet entirely known. For example, smelling fruity odorants (olfactory modality) is found to increase the sweetness perception (gustatory) of food. Thus, one could imagine that a fruity odorant produced in the e-cigarette may also increase the sweetness of the PG/VG solvent or other sweet tastes within the e-cigarette. Taken together, the above reviews illustrate that flavors do not act in isolation, but rather, in the context of not just the solvent they are dissolved but also additional flavor interactions that merit further study.

## Summary

Collectively, these studies point to a more nuanced understanding of how flavors can alter the tobacco use experience thereby contributing to a rise in nicotine intake. On the one hand, there appears to exist genetic and environmental factors that control preference and masking ability as illustrated for menthol. This likely holds true for other flavors, and should be further explored by the scientific community. On the other hand, there appears to be the potential for a great number of flavor-flavor or flavor-solvent interactions that can contribute to the overall sensory experience of tobacco product use. Identifying shared interactions among flavors and solvents on overall tobacco product use will be important to simplify this problem.

## Author contributions

RW wrote topic summary and Editorial. AJ, BH, and NK reviewed the Editorial. All authors contributed to the article and approved the submitted version.

## Funding

AJ was funded by Food and Drug Administration 1K01DA051882-01.

## Conflict of interest

The authors declare that the research was conducted in the absence of any commercial or financial relationships that could be construed as a potential conflict of interest.

## Publisher's note

All claims expressed in this article are solely those of the authors and do not necessarily represent those of their affiliated organizations, or those of the publisher, the editors and the reviewers. Any product that may be evaluated in this article, or claim that may be made by its manufacturer, is not guaranteed or endorsed by the publisher.
